# Chronic diseases: what about infections of virus and prions *via* the gut?

**DOI:** 10.1177/17562848211028805

**Published:** 2021-09-28

**Authors:** Helge Waldum, Tom Christian Martinsen

**Affiliations:** Department of Clinical and Molecular Medicine, Faculty of Medicine and Health Sciences, Norwegian University of Science and Technology, Jakobstien 4, Trondheim, 7021, Norway; Department of Clinical and Molecular Medicine, Faculty of Medicine and Health Sciences, Norwegian University of Science and Technology, Trondheim, Norway; St. Olavs Hospital, Trondheim University Hospital, Trondheim, Norway

**Keywords:** gastric acid, intestinal bacteria, intestinal microbiology

Dear Editor,

The gut bacterial microbiome has been studied extensively and quantitative differences have been attributed to different conditions and diseases. However, interest has been concentrated on bacteria and no other microorganisms. During the last decade methodological improvements have made it possible to study virus and other microorganisms.^1–3^ Since the description of Mad Cow Disease being transmitted to man by food,^4,5^ we have wondered about the lack of interest in gut virus and prions by the medical community.^6^ In spite of the lack of knowledge of the aetiology of chronic infectious disease like inflammatory bowel disease, so-called neurodegenerative diseases and most cancers, few if any have been concerned about the risk of installing faeces into the gut to treat irritable bowel syndrome,^7^ or even neurological diseases,^8^ based on a belief that only known bacterial pathogens could play any pathogenic role and that observations for a few months could secure safety. Taking into consideration the known latency between infection and development of cancer as well as prion diseases,^9,10^ such a view reflects a naivety and lack of biological knowledge. In fact, one of us wrote a paper 40 years ago that most of the chronic inflammatory and neoplastic diseases in the gastrointestinal tract were of viral origin.^11^ Recently, the possible role of virus in chronic inflammatory bowel disease as well as gastrointestinal cancer, including colonic cancer, has been discussed.^12–14^ Many years ago, we published our papers on the role of gastric juice in the defence against prion diseases,^15,16^ without attracting any interest at all. In general, the role of normal gastric juice in the defence against infections including virus has not been focussed on, although viruses having the property to escape destruction by gastric juice were classified as enterovirus (poliovirus belongs to this group). Together with the proteolytic enzyme pepsin, and possibly also the gastric lipase, the gastric acid is central to the destruction of microbes.^17^ Use of inhibitors of gastric acid secretion will accordingly reduce the ability of gastric juice to kill swallowed microorganisms and possibly by this mechanism increase the risk of diseases with unknown aetiology, including cancers. Use of proton pump inhibitors (PPIs) has been claimed safe from observation studies of a few years without discussing these aspects,^18^ and an accompanying leader declared that the question of PPI toxicity was solved.^19^

We hope that the focus on the virome will lead to increased caution and vigilance when it comes to use of inhibitors of gastric acid secretion as well as installation of faeces in the gut.
